# Transferrin-Conjugated Docetaxel–PLGA Nanoparticles for Tumor Targeting: Influence on MCF-7 Cell Cycle

**DOI:** 10.3390/polym11111905

**Published:** 2019-11-19

**Authors:** Sajan Jose, Thomas A. Cinu, Rosmy Sebastian, M. H. Shoja, N. A. Aleykutty, Alessandra Durazzo, Massimo Lucarini, Antonello Santini, Eliana B. Souto

**Affiliations:** 1 Department of Pharmaceutical Sciences, Mahatma Gandhi University, Cheruvandoor Campus, Ettumanoor 686631, India; cinusaj@gmail.com (T.A.C.); rosmysebastian2009@gmail.com (R.S.); 2 CEB-Centre of Biological Engineering, University of Minho, Campus de Gualtar, 4710-057 Braga, Portugal; 3 College of Pharmaceutical Sciences, Manipal University, Manipal 576104, India; shojamh@gmail.com; 4 Caritas College of Pharmacy, Kottayam 686630, India; aleykuttyna@gmail.com; 5 CREA—Research Centre for Food and Nutrition, Via Ardeatina 546, 00178 Rome, Italy; alessandra.durazzo@crea.gov.it (A.D.); massimo.lucarini@crea.gov.it (M.L.); 6 Department of Pharmacy, University of Napoli Federico II, Via D. Montesano 49, 80131 Napoli, Italy; 7 Department of Pharmaceutical Technology, Faculty of Pharmacy, University of Coimbra (FFUC), Pólo das Ciências da Saúde, 3000-548 Coimbra, Portugal

**Keywords:** transferrin conjugate, tumor targeting, docetaxel trihydrate, PLGA nanoparticles, factorial design

## Abstract

Targeted drug delivery systems are commonly used to improve the therapeutic index of anti-cancer drugs by increasing their selectivity and reducing systemic distribution and toxicity. Ligand-conjugated nanoparticles (NPs) can be effectively applied for active chemotherapeutic targeting to overexpressed receptors of tumor cells. In this study, transferrin (T*f*) was successfully conjugated with poly-l-lactic-co-glycolic acid (PLGA) using ethylene diamine confirmed by NMR, for the loading of docetaxel trihydrate (DCT) into PLGA nanoparticles (NPs). The DCT-loaded T*f*-conjugated PLGA NPs were produced by an emulsion-solvent evaporation technique, and a 3^2^ full factorial design was used to optimize the nanoparticle formulations. The DCT-loaded T*f*-conjugated PLGA NPs were characterized by FTIR spectroscopy, differential scanning calorimetry, powder X-ray diffraction (PXRD), TEM, particle size, and zeta potential analysis. In vitro release kinetics confirmed that release of DCT from the designed formulations followed a zero-order kinetics and a diffusion controlled non-Fickian release profile. The DCT-loaded T*f*-conjugated PLGA NPs were evaluated in vitro in MCF-7 cells for bioactivity assessment. Cytotoxicity studies confirmed that the T*f*-conjugated PLGA NPs were more active than the non-conjugated counterparts. Cell uptake studies re-confirmed the ligand-mediated active targeting of the formulated NPs. From the cell cycle analysis, the anti-cancer activity of DCT-loaded T*f*-conjugated PLGA NPs was shown to occur by arresting the G_2_/M phase.

## 1. Introduction

Chemotherapy is traditionally used for cancer treatment; however, its therapeutic efficacy is usually limited by two major challenges. Firstly, there is the risk of occurrence of multidrug resistance (MDR) phenotypes leading to the unsuccess of chemotherapy. In fact, the major mechanism of MDR has been attributed to the *MDR1* gene which codifies for the P-glycoprotein (P-gp) in cancer cells [1]. Secondly, chemotherapeutic drugs can unselectively enter into both healthy and tumor tissues, resulting in undesirable side effects and even death of the patients. Significant efforts have therefore been made to develop alternative therapies that improve the therapeutic index of anticancer drugs both by increasing their efficiency and decreasing their toxicity [2,3]. 

In recent years, the design and synthesis of biocompatible and biodegradable nanoparticles have opened new perspectives for several biological and biomedical applications [4,5,6,7,8,9]. Among them, polymeric nanoparticles have emerged as promising carriers for targeting poorly water-soluble or amphiphilic drugs [4,5,6] as well as genes to tumor tissues [7,8]. The vasculature in tumors is leaky to macromolecules, and the tumor lymphatic system is usually deficient, thus nanoparticles (NPs) can preferentially be delivered into the tumor through the enhanced permeation and retention (EPR) effect via its blood vessels [9]. Still, it was found that polymeric NPs could reduce the multidrug resistance by a mechanism of internalization of the drug and reducing its efflux from cells mediated by P-gp [10,11]. However, it is of critical importance to develop a more specific and active delivery system that could target the tumor and enhance intracellular uptake of the drug to the tumor site. Selective interactions set between cancer cell receptors and specific targeting moieties decorating the surface of nanoparticles have been exploited. Some ligands, such as folate [12,13,14,15] and transferrin [16,17], can be conjugated to the polymer back-bone and substantially increase site-specific targeting of drug loaded NPs. A synergistic combination of dual-targeting ligands has also been proposed to enhance in vitro and in vivo tumor targeting [18].

The major challenge in the active targeting using nanoparticles is the development of drug/gene loaded nanoformulation containing a conjugated ligand or antibody. The complexity of the formulation development, stability of the formulation and difficulty in scaling up are the reasons for very little marketed products of this kind [19]. There is thus an urgent need for developing simpler and newer techniques for tumor targeted delivery of anticancer drugs. On the other side, clinical trials with nanomedicines, in Europe, have increased; studies on follow-up, use, and compliance, as reported by recent studies in the area [20,21,22] as well as communication strategies and assessment [23,24] are needed. 

In this study, we propose a novel approach based on transferrin (Tf)-conjugated poly(lactide-co-glycolide) (PLGA) nanoparticles loaded with docetaxel trihydrate (DCT) for tumor targeting. Poly(lactide-co-glycolide) (PLGA) was selected as a polymer matrix because it is a biodegradable copolymer widely used in many Food and Drug Administration (FDA)-approved drug formulations. The PLGA-NPs have also been reported to be appropriate for the loading or poorly water-soluble drugs for parenteral and ocular administration [4,5,6,25,26,27,28,29,30]. The glycoprotein transferrin was selected as a ligand because it is upregulated on the surface of cancer cells. The increased iron requirement in cancer cells results in higher expression of transferrin receptors in these cells compared to the normal ones. Docetaxel trihydrate (DCT) is a second generation taxane derived from a compound found in the European yew tree *Taxus baccata* [31]. The drug is practically insoluble in water and is being currently used in chemotherapy of gastro/esophageal [32,33,34,35,36] and breast [37,38,39,40] cancers. It binds precisely to the β-tubulin subunit of microtubules and antagonizes the disassembly of this key cytoskeletal protein, with the result that bundles of microtubules and aberrant structures, derived from the microtubules, appear in the mitotic phase of the cell cycle. Arrest in the mitosis follows.

We report for the first time the use of a 3^2^ full factorial design for the optimization of Tf-conjugated PLGA NPs for the loading of DCT, produced by a modified oil-in-water (o/w) emulsion solvent evaporation technique [29,41]. The factorial design generally depends on first degree mathematical models. Full factorial designs involve studying the effect of all the factors at various levels, including the interactions among them. The mathematical model associated with the design consists of the main effects of each variable plus all the possible interaction effects among factors in the model [42]. The PLGA–EDA–transferrin conjugate was synthesized according to a procedure optimized in our lab. The release profile has also been characterized using several mathematical models, namely, zero- and first-order kinetics, Higuchi, and Korsmeyer–Peppas.

## 2. Materials and Methods

### 2.1. Chemicals

Docetaxel trihydrate was obtained as a gift sample from Mac Chem Products Pvt Ltd. (Mumbai, India). The PLGA (50:50), polyvinyl alcohol (PVA), coumarin-6, and human transferrin (T*f*) were purchased from Sigma–Aldrich (Bangalore, India). Dichloromethane (DCM), ethylene diamine, and dimethyl sulfoxide (DMSO) were purchased from Merck Chemicals (Mumbai, India). N-Hydroxy succinimide (NHS) and 1-ethyl-3(3-dimethyl aminopropyl) carbodiimide (EDC) were purchased from Himedia (Mumbai, India). All other reagents used were of analytical grade and home supplied.

### 2.2. Synthesis of PLGA–EDA–Transferrin Conjugate and NMR Analysis

**Figure 1:**
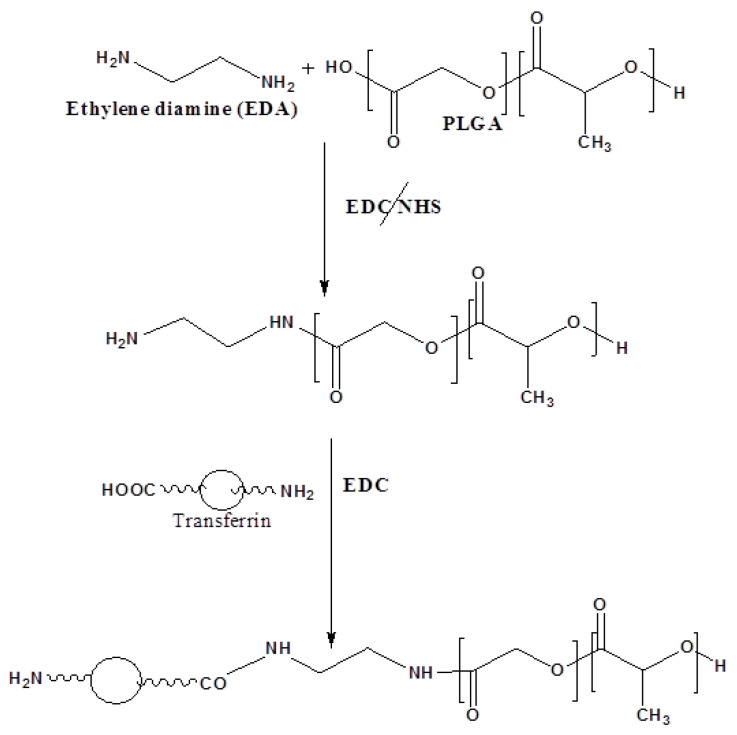
Synthesis of the poly(lactide-co-glycolide) (PLGA)–EDA–transferrin conjugate.

Prior to the production of nanoparticles, the PLGA–EDA–transferrin conjugate was synthesized according to Figure 1. The selected proportions of reactants were based on preliminary studies to optimize the total use of ligand in the produced number of nanoparticles.

A solution of PLGA (0.01 mmol), 1-ethyl-3-(dimethylaminopropyl) carbodiimide (EDC) (0.08 mmol), and *N*-hydroxysuccinimide (NHS) (0.08 mmol) was prepared in a round-bottom flask using anhydrous DCM as solvent and left to stir overnight. The obtained solution was then filtered and added to cold anhydrous ether dropwise to allow the precipitation of activated PLGA. Ether was removed by decantation and the obtained polymer was then dried overnight under vacuum to obtain the solid activated PLGA. The obtained solid activated polymer (0.01 mmol) was dissolved in 8 mL anhydrous DCM in a round-bottom flask. An amount of 0.05 mmol of ethylene diamine EDA was dissolved in 2 mL anhydrous DCM and added to the freshly prepared polymeric solution. The mixture was then gently stirred at 400 rpm under nitrogen atmosphere for 6 h. The reaction mixture was added dropwise to cold anhydrous ether to precipitate the product which was dried under vacuum. To wash the un-reacted EDA, the product was dissolved in DMSO and transferred into a dialysis bag. The content of the bag was dialyzed against 5 L of de-ionized water for 48 h at 4 °C with constant stirring at 600 rpm (with 4 changes). The obtained dispersion was freeze-dried and stored at a temperature of −20 °C. Transferrin (0.00125 mmol) was activated with EDC (0.0025 mmol) and NHS (0.0025 mmol) using anhydrous DMSO in the presence of triethylamine (0.1 mL), under atmosphere protected from light. The by-product, dicyclohexylurea, was removed by filtering the solution and activated transferrin was precipitated in cold anhydrous ether. The product was washed several times, decanted, and dried. Activated T*f*, EDC (0.006 mmol) and PLGA–EDA (0.024 mmol) were co-dissolved in anhydrous DMSO in light protected conditions for 8 h. The EDC was added to ensure that all the transferrin is ready to react with PLGA–EDA. An excess amount of activated transferrin was used to achieve a higher conjugation of T*f* to PLGA–EDA. The obtained product was filtered, precipitated using methanol as solvent, followed by drying under vacuum. To precipitate free un-reacted transferrin, the dried product was dissolved in DCM, followed again by filtration. The organic solvent (DCM) present in the solution was then removed by evaporation under vacuum. The obtained product was analyzed by proton NMR spectroscopy on a Bruker AV 400–400 MHz High resolution Multinuclear FT-NMR spectrometer (Bruker BioSpin, Rheinstetten, Germany).

### 2.3. Formulation of Drug-Loaded Nanoparticles Using Polymer Conjugates

The transferrin (T*f*) conjugate PLGA nanoparticles loaded with docetaxel trihydrate (DCT-T*f*-PLGA NPs) were prepared by a modified oil-in-water (o/w) emulsion solvent evaporation technique [43]. The ligand conjugated polymer was accurately weighed and dissolved in dichloromethane (DCM) and 5 mg drug in 0.1 mL DMSO. Drug solution was added with gentle stirring to the polymer solution to dissolve the contents. This organic phase was added slowly to aqueous phase containing poly vinyl alcohol (PVA) as stabilizer and sonicated using a probe sonicator (Sonics Vibracell, USA) at an output of 40W in an ice bath. The o/w emulsion formed was gently stirred at room temperature by a magnetic stirrer (Tarson, Mumbai, India) for up to 12 h for complete evaporation of organic solvent. The resulting suspension was centrifuged at 15,000 rpm for 20 min at 4 °C to separate the nanoparticles. The particles were washed with distilled water, thrice, to remove the emulsifier and adsorbed drug molecules. Then, particles were freeze-dried for 24 h using a lyophilizer (Sub-Zero, Chennai, India) at −85 °C and 0.001 mbar pressure.

### 2.4. Optimization of the Formulation Process

The DCT-loaded T*f*-conjugated PLGA NPs of desired quality is affected by different factors. Optimizing these factors by trial and error method is tedious and costly. Design of experiments (DoE) was therefore applied for the optimization of the process of nanoparticle formulation with the help of Design Expert ^®^ ver. 8.0.7.1 software (Statease, Minneapolis, USA). A two-factor three-level full factorial design was used in the optimization of nanoparticles for the study of the influence of the two independent variables on the responses (Y_1_ and Y_2_) particle size and percentage of encapsulated drug. The DoE was therefore appropriate for the study of the quadratic surface responses and for the construction of the second order polynomial models. After measuring the responses with either simple linear (Y= X_0_ + X_1_A + X_2_B), interactive (Y= X_0_ + X_1_A + X_2_B + X_5_AB) or quadratic (Y = X_0_ + X_1_A + X_2_B + X_3_A_2_ + X_4_B_2_ + X_5_AB + E) models, the values of selected variables at different levels can be obtained by multiple regression analysis of the data and F statistics to identify the statistically significant terms. The reduced equation (i.e., an equation based solely on statistically significant terms) is then used for drawing contour plots to evaluate the influence of selected variables when changing from low to high level. The non-linear quadratic model generated by the design is in the form Y = X_0_ + X_1_A + X_2_B + X_3_A_2_ + X_4_B_2_ + X_5_AB + E, where Y is the measured response associated with each factor level combination: X_0_ is an intercept; X_1_–X_5_ are the regression coefficients; A and B are the factors studied; and E is the associated error term.

Based on the pre-optimization studies, three square full factorial designs were selected for optimization. The phase ratio and sonication time were found to have significant effect on particle size and encapsulation efficiency. The design matrix was fixed, and three levels were chosen based on the data from the pre-optimization. While the success of the formulation depends on the particle size and encapsulation efficiency, these were therefore chosen as the responses. The three-square design matrix and the factors with their levels are shown in Table 1. The responses (i.e., particle size and encapsulation efficiency) were statistically evaluated using Design Expert 8.0.7.1 software (Stat-Ease Inc., Minneapolis, USA).

### 2.5. Determination of Encapsulation Efficiency and Drug Loading Capacity

The encapsulation Efficiency (EE%) and the loading capacity were determined by a direct method. Briefly, accurately weighed 5 mg of the freeze-dried nanoparticles were vortexed with 2 mL of DCM for 1 h and filtered through 0.22 µm membrane filter. The drug content in the filtrate was then analyzed by HPLC. The percentage of drug encapsulation, a measure of the encapsulation efficiency, was calculated as the ratio of the drug content in the freeze-dried powder and the initial drug amount added for the production [29]. The drug loading capacity was determined as the ratio of the drug content to the freeze-dried powder.

(1) $$\mathrm{Encapsulation}\;\mathrm{efficiency}\;(\%)\;=\frac{\mathrm{Loaded}\;\mathrm{drug}}{\mathrm{Total}\;\mathrm{drug}\;\mathrm{added}}\;\times 100$$

(2) $$\mathrm{Loading}\;\mathrm{capacity}\;(\%)\;=\frac{\mathrm{Loaded}\;\mathrm{drug}}{\mathrm{Total}\;\mathrm{amount}\;\mathrm{of}\;\mathrm{polymer}}\;\times 100$$

### 2.6. Fourier Transform Infrared Spectroscopy (FTIR)

The FTIR was used to analyze freeze-dried DCT-loaded T*f*-conjugated PLGA NPs to study the chemical properties of docetaxel trihydrate and drug-loaded nanoparticles by functional group analysis. The samples were analyzed by an FTIR Spectrum 400 analyzer (Perkin Elmer, Massachusetts, USA) and reported in wave number (cm^−1^). The scanning range was 400–4000 cm^−1^. 

The FTIR spectroscopy can be defined as a “*fingerprint* analytical technique” for the structural identification of compounds considering that no two chemical structures will have the same FTIR spectrum. The FTIR provides a characteristic signature of chemical or biochemical substances present in the sample by featuring their molecular vibrations (stretching, bending, and torsions of the chemical bonds) in specific infrared regions [44].

### 2.7. Differential Scanning Calorimetry (DSC)

Differential scanning calorimetry was used to analyze bulk PLGA, transferrin, docetaxel trihydrate, and their physical mixture as well as the freeze-dried DCT-loaded T*f*-conjugated PLGA NPs to compare the thermograms. The analysis was carried out in a DSC from TA Instruments (MDSC 2910, USA).

### 2.8. Powder X-ray Diffraction (PWRD)

Powder X-ray diffraction patterns of bulk PLGA, transferrin, docetaxel trihydrate, and their physical mixtures as well as the freeze-dried DCT-loaded T*f*-conjugated PLGA NPs were compared to study the change in crystal structure using X-ray diffractometer X’pert PRO, (PANalytical, Almelo, The Netherlands).

### 2.9. Transmission Electron Microscopy (TEM)

Transmission electron microscopy was used to obtain more detailed surface images of nanoparticles. Analysis was performed in a JEOL JEM-1010 electron microscope (JEOL Ltd., Tokyo, Japan). The nanoparticles were suspended in Milli-Q water. A tiny drop of sample was pipetted onto the parafilm using a micropipette, placing the shiny side of the TEM grid on the drop and left for 20 min for the particles to adsorb onto the grid. The grid was then removed and placed on a tissue paper (Kimwipe without fibers) with the shining side up for 1 h for drying. Image scanning of the sample was performed under different magnifications.

### 2.10. Mean Particle Size, Size Distribution, and Zeta Potential

The mean particle size, size distribution, and zeta potential (ZP) of the optimized surface-modified nanoparticles were determined by dynamic light scattering technique using Zetasizer Nano ZS, nanoseries, Malvern Instruments, MA, USA). A concentration of 0.1% by weight of the sample was prepared in phosphate buffered solution (PBS) of pH 7.4 for zeta potential measurement. 

### 2.11. In Vitro Drug Release Studies

The drug release profile of docetaxel from PLGA nanoparticles was studied using a dialysis technique. Briefly, a mass of freeze-dried nanoparticles equivalent to 5 mg of docetaxel was weighted, dispersed in 1 mL of phosphate buffer solution at pH 7.4 and placed in a dialysis bag (Spectra/Por^®^, molecular weight cut-off 12,000 Da). The dialysis bag was sealed at both ends, soaked in 100 mL of phosphate buffer solution (pH 7.4), and maintained at 37 ± 0.5 °C and at 100 ± 5 rpm in a shaker. Sampling volumes were taken at predetermined time intervals, being replaced by the same volume of fresh phosphate buffer solution to keep sink conditions. The amount of docetaxel trihydrate released into the medium was quantified by HPLC and compared to a generated standard calibration curve [45]. The experiment was done in triplicate and results are expressed as the mean and standard deviation.

### 2.12. In Vitro Bioactivity Studies

The cytotoxicity of nanoparticles was evaluated using the MTT assay in MCF-7 cells. The MCF-7 cells (human breast cancer cell line) were obtained from NCCS Pune, India. Cells were grown in 25 cm^2^ tissue culture flasks containing Dulbecco’s modified Eagle’s medium (DMEM) supplemented with 10% FBS, 1% l-glutamine, and 50 μg/mL gentamycin sulphate. Cell uptake studies were carried out with coumarin-6-tagged nanoparticles by flow cytometric determination [46] and cell cycle analysis was also done with MCF-7 cells by flow cytometry [47,48].

#### 2.12.1. MTT (3-(4, 5-Dimethylthiazol-2-yl)-2, 5-Diphenyltetrazolium Bromide) Assay

Exponentially growing cell lines were harvested from a 25 cm^2^ tissue culture flask, and a stock cell suspension (5 × 104 cell/mL) was prepared. A 96 well flat bottom tissue culture plate was seeded with 5 × 103 cells in 0.1 mL of DMEM supplemented with 10% FBS and allowed to attach for 24 h. Test formulations were freshly prepared prior to the experiment and serially diluted with medium to obtain the desired concentrations. After 24 h of incubation, cells were treated with 100 µL of test formulation and incubated for 24 h. Each treatment was performed in triplicate. At the end of each experiment, medium was removed and washed with 200 µL of PBS. To each 96 well plate, 100 µL of MTT reagent (stock: 1 mg/mL in serum free medium) was added and incubated at 37 °C for 4 h. After 4 h of incubation, the plate was blotted on tissue paper to remove the MTT reagent. To solubilize formazan crystals in the wells, 100 µL of 100% DMSO was added to each well, and the optical density was measured at 540 nm. 

#### 2.12.2. Estimation of Coumarin-6 Tagged Nanoparticles Uptake by Flow Cytometry 

The MCF-7 (1 × 10^6^) cells were seeded in 96 well plates and incubated at 37 °C for 24 h in a humidified CO_2_ incubator. After 24 h of incubation, the medium was aspirated from the plates, and cells were washed with DMEM. A volume of 10 µL of nanoparticles conjugated with coumarin-6 (at their respective IC_50_ concentrations) were incubated at 37 °C in the CO_2_ incubator (for 2 h and 24 h). The formulation in the medium was then aspirated, cells were washed thrice with PBS and scraped in 1 mL of fresh PBS using a cell scraper. The cells were analyzed using Accuri C6 flow cytometer (BD Biosciences, San Jose, CA, USA) excitation at 488 nm and emission at 533/30. A minimum of 10,000 events were recorded and analysis of flow cytometric data was performed using BD software. The first gating was done for a single cell population selection on a forward (FSS) versus side scatter (SSC) dot plot. The second dot plot was FL-1-A versus FL-4-A (533/30 versus 675/25) for selection of coumarin-6 fluorescence in the FL-1 channel. Plain normal cells were used for the normalization of the internal fluorescence or auto-fluorescence. The shift in mean fluorescence in FL1-A versus count plot was calculated [48].

#### 2.12.3. Cell Cycle Analysis

The MCF-7 (1 × 10^6^) cells were seeded in 25 cm^2^ flasks and after overnight adherence, incubated for 24 h with test formulations at their respective IC_50_ concentrations. Trypsinization was used to detach the cells followed by mixing with floating cells, centrifuged and PBS washed. The obtained cell pellets were fixed in 70% ice-cold methanol for 24 h at −20 °C. Cell pellets were washed with PBS and isotonic PI solution (25 µg/mL propidium iodide, 0.03% NP-40 and 40 µg/mL RNase A) was added. The Accuri C6 flow cytometer (BD Biosciences, San Jose, CA, USA) was used for the analysis of stained cells, applying 488 nm excitation and emission at 575/40 nm. A minimum of 10,000 events were recorded for each sample, and data analysis was done using BD Accuri™ C6 software [49,50].

## 3. Results and Discussion

**Figure 2:**
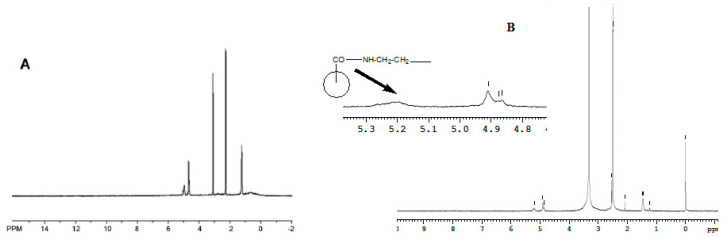
(**A**) ^1^H NMR spectrum of PLGA (**B**). ^1^H NMR spectrum of transferrin-conjugated PLGA–EDA.

The PLGA–EDA–transferrin conjugate was synthesized according to the procedure optimized in our lab (Figure 1). The carboxy-terminal end group of PLGA was activated with 1-ethyl-3-EDC and linked to n-boc-ethylenediamine via an amide bond which was then de-protected to obtain PLGA–EDA with a free amine group. The water-soluble carbodiimide heterobifunctional crosslinker EDC is commonly used to couple carboxyl groups to primary amines. The ^1^H and ^13^C NMR are commonly used for the characterization of new compounds. In this work, we used ^1^HNMR for which the spectra of PLGA and PLGA–EDA–transferrin are shown in Figure 2. 

The characteristic peaks confirming the successful conjugation were found in the ^1^HNMR spectra of PLGA–EDA–transferrin conjugate. In Figure 2B, the peaks from 1.23 to 1.47 ppm were attributed to methyl hydrogens (–CH_3_) of PLGA segments, identified as (^|^). The peaks at 4.86 and 4.91 (identified as (^|^) in Figure 2B) were assigned to protons of hydroxyl and methine groups, respectively. The peaks at 2.541 ppm and 2.501 (identified as (^|^) in Figure 2B) belonged to the methylene hydrogen groups of EDA and transferrin segments. Although weak, the peak recorded at 5.2 confirmed the –CONH– bond formation between the C-terminal of transferrin and –NH_2_ group of EDA (Figure 2B). The recorded spectrum demonstrates the successful conjugation of PLGA, EDA, and transferrin.

The procedure selected for the production of transferrin-conjugated PLGA nanoparticles was based on the solubility properties of docetaxel trihydrate. A modified oil-in-water (o/w) emulsion solvent evaporation technique was used, based on the emulsification of an organic solution of polymer in dichloromethane (DCM) (containing 5 mg drug in 0.1 mL DMSO) in an aqueous phase followed by evaporation of the organic phase. The DCT was practically insoluble in water but showed approximately 5 mg/mL solubility in DMSO being therefore retained in the inner phase of the emulsion. The o/w emulsion was produced by processing the inner phase in the outer phase by high-shear homogenization. The organic phase was evaporated by stirring overnight in a magnetic stirrer. The nanoparticles were collected by ultracentrifugation and washed with Milli-Q water to remove any surfactant or free drug. The resulting nanoparticles were lyophilized to obtain free flowing drug-loaded PLGA nanoparticles. The PLGA NPs showed superior advantages over other drug carriers (e.g., liposomes, solid lipid nanoparticles), as the poorly water-soluble drug can be covalently linked to the carrier (as demonstrated in Figure 1).

Three square design is a two-factor three-level design, originating 13 runs that were used in this experiment. The two factors selected were the phase ratio and sonication time. An organic-to-aqueous ratio of 1:4 and sonication time of 8 min were selected as the central point in the 3^2^ full factorial design. Based on the results obtained during pre-formulation testing, the amount of docetaxel trihydrate in the formulation was fixed as 5 mg and of ligand-conjugated polymer as 10 mg. The sonication amplitude was kept at 40 W. The effect of the independent variables (phase ratio and sonication time) on the mean particle size and EE% of the 13 produced PLGA NPs is given in Table 2.

Thirteen formulations (1-Tf-PLGA NPs to 13-Tf-PLGA NPs) were prepared according to the set of experiments defined in the design matrix (Table 1). Polynomial equations for the individual main effects and interaction factors were obtained for each of the individual responses (mean particle size and EE%) based on the multiple correlation coefficient (R^2^), adjusted multiple correlation coefficient (adjusted R^2^) and predicted residual sum of squares (PRESS). The experimental data was fit to four high degree polynomial models viz. linear, interactive (2FI), quadratic and cubic models (Table 3). Three different tests, i.e., the sequential model sum of squares, lack of fit tests, and model summary statistics, were applied to predict the adequacy of the models which represent the minimum particle size. The prob > F value of *p* < 0.0001, low standard deviation, high *R*-squared, and lower predicted residual error sum of square (PRESS) value suggested to select the quadratic model for the response particle size (Y1). The suggested model for the response encapsulation efficiency (Y2) was the linear model. The model analysis data of the response particle size and encapsulation efficiency are given in Table 4.

The model F-value of 54.57 for the response Y_1_ and 157.99 for the response Y_2_ translates the significance of the model. Because of the magnitude of the model “F-value”, there was only a 0.01% chance to occur due to the noise. Values of “Prob > F” lower than 0.0500 stand for the significance of the model terms (i.e., A, B, AB, A^2^) were significant model terms for the response Y_1_, whereas A and B are significant model terms for the response Y_2_. The model terms were not significant when the values were greater than 0.1000. “Lack of Fit” was not significant with respect to the pure error if the “Lack of Fit F-value” were 0.31 and 2.68 for the responses Y_1_ and Y_2_, respectively. There was an 82.11% chance for the response Y_1_ and a 17.96% chance for the response Y_2_ for the “Lack of Fit F-value” of this large to occur due to the noise. The lack of fit was shown not to be significant. The polynomial equation for the measured responses was obtained with the statistical software, generating the following equations for each of the responses:

Particle size (Y_1_) = 254.39 − 75.68A − 31.65B + 22.35AB + 37.97A^2^ + 2.17B^2^

Encapsulation (Y_2_) = 45.24 − 10.45A − 3.78B

**Figure 3:**
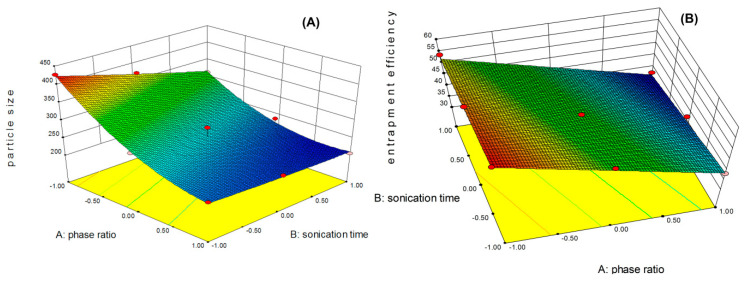
The 3D response surface plot of the responses for particle size Y1 (**A**) and encapsulation efficiency Y2 (**B**).

The equations represent the quantitative effect of variable (A, B) and their interactions on the responses. Coefficients with more than one factor term and those with higher order terms represent interaction terms and quadratic relationships, respectively. A synergistic effect is represented by a positive trend while an antagonistic effect is represented by a negative trend. The interactions between factors and the responses were further studied by using the contour plot and the 3D response surface plot (Figure 3). It is evident from the plot that both particle size and encapsulation efficiency decreased as the aqueous-to-oil phase ratio increased. Similarly, an increase in the sonication time also led to a decrease in the particle size, as well as the encapsulation efficiency, but the effect was slightly lower when compared to the phase ratio.

Based on the results obtained from the DoE, the formulations selected for the preparation of drug-loaded nanoparticles are given in Table 5.

The above levels of factors (Table 5) were used for the production of an optimized formulation. The amount of dodecyl trihydrate added to the formulation was 5 mg, the amount of polymer was fixed to 10 mg, PVA concentration was 2%, and sonication amplitude used was 40 W. The DCT-loaded T*f*-conjugated PLGA NPs depicted a mean particle size of 210.6 ± 2.7 nm (polydispersity index (PDI) of 0.131 ± 0.021), while DCT-loaded PLGA NPs showed a mean size of 183 ± 2.4 nm and PDI of 0.027 ± 0.003.

The FTIR spectrum of the drug-loaded nanoparticles were compared to that of the physical mixture of docetaxel trihydrate and polymer (Supplementary Materials Figure S1). All the characteristic peaks of docetaxel trihydrate and PLGA–EDA–transferrin conjugate were present in the IR spectrum of the nanoparticles. An additional small peak at 1709 cm^−1^ was also recorded and was attributed to the formation of an amide bond between PLGA with EDA and EDA with transferrin during the conjugation process.

The prepared nanoparticles were analyzed by DSC in order to understand the physical status, in comparison to the bulk counterparts (Supplementary Materials Figure S2). The thermal analysis was considered as a tool for examining whether the solute particles were dispersed well in the polymeric matrices. The DSC thermogram of docetaxel-loaded nanoparticles exhibited endothermic peaks at 48 °C and 93 °C, attributed to the presence of PLGA and transferrin, respectively. The endothermic peak in the range of 165 °C to 175 °C, which was previously observed in the independent thermogram of DCT and its physical mixture, was not observed in the nanoparticles. The absence of the DCT peak in the nanoparticle formulation confirms that drug molecules were dispersed in the polymeric network to form a homogenous matrix.

To study the interaction between drug and polymer and the degree of sample crystallinity, the results of the PXRD analysis of DCT-loaded T*f*-conjugated PLGA NPs were compared to those obtained for the physical mixture of drug and polymers. The characteristic peaks of DCT at 2θ angles of 10.3, 11.1, 14.2, 17.8, 19.8, and 22.2° were present in the physical mixture, which indicates the crystalline nature of the drug DCT (Supplementary Materials Figure S3A and S3B). The DCT-loaded T*f*-conjugated PLGA NPs showed no sharp peaks in the DSC thermogram (Supplementary Materials Figure S3C). This translates the amorphous and disordered-crystalline phase of the drug within the polymeric matrix of the NPs or its presence in the form of molecular dispersion.

**Figure 4:**
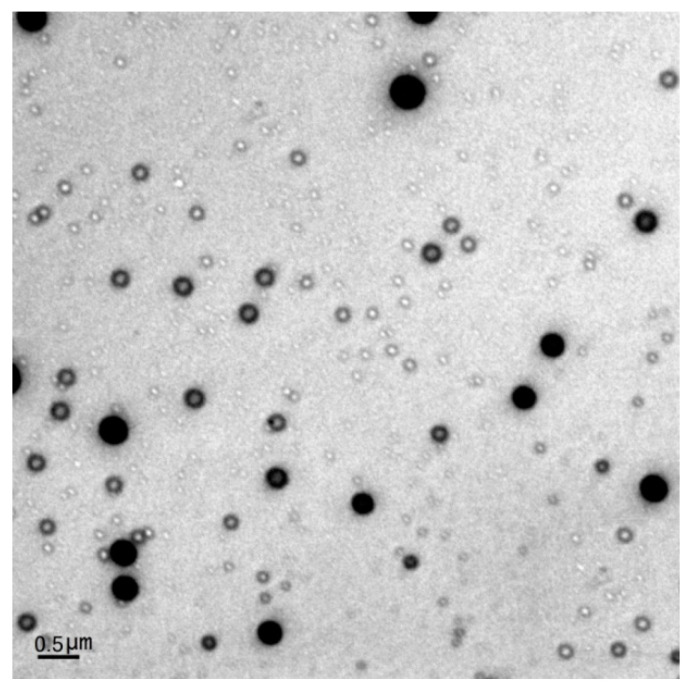
TEM image of DCT-loaded T*f*-conjugated PLGA NPs.

The shape and surface morphology were analyzed by TEM. Surface morphology analysis showed PLGA nanoparticles of homogenous, smooth, and spherical shape, discrete, and of a uniform size distribution (Figure 4), while freeze-drying did not cause much nanoparticle aggregation (data not shown). Figure 4 depicts some droplets of organic solvent (DMSO) which corresponded to the inner phase used in the production of the nanoparticles by the solvent evaporation technique. This was attributed to the incomplete evaporation of DMSO, after which PLGA NPs should depict a dense, continuous polymeric network (as exhibited by the darker NPs). Important to note is the absence of drug recrystallized in the aqueous phase while the spherical shape of NPs ensures minimum segregation effect.

The optimized nanoparticles were evaluated for particle size and zeta potential, and the results are shown in Supplementary Materials Figure S4A and S4B. The mean particle size was found to be 210.6 ± 2.7 nm and the zeta potential approximately −24.5 mV. While the literature recommends a minimum zeta potential of ±40 mV for nanosuspensions to show stability solely by electrostatic repulsion, according to the DLVO theory, the obtained zeta potential values for the produced nanoparticles contributed for their physical stability on the shelf-life. 

**Figure 5:**
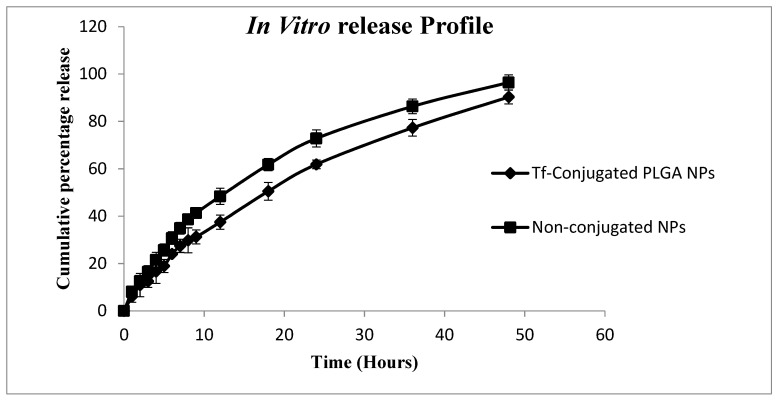
In vitro release profile of DCT-loaded T*f*-conjugated PLGA NPs and unconjugated nanoparticles.

The in vitro release profiles of DCT-loaded T*f*-conjugated PLGA NPs were obtained by suspending the prepared nanoparticles in phosphate buffer pH 7.4 and quantifying the DCT solubilized in the medium. The release of DCT from T*f*-conjugated PLGA NPs for a period of 48 h was determined using the HPLC method [49]. The cumulative percentage release data of DCT was plotted against time, and it was compared to the unconjugated DCT-loaded nanoparticles (Figure 5). The cumulative percentage of drug release of T*f*-conjugated PLGA NPs (90.31% ± 2.98%) was found to be lower than unconjugated NPs (96.42% ± 3.24%) at the end of 48 h. This result was attributed to the increase in the structural complexity of the polymer due to the transferrin conjugation.

The modified release profile of both T*f*-conjugated PLGA NPs and non-conjugated NPs anticipate that the drug was indeed within the polymeric matrix which modulates its release. These results corroborate those obtained from the ^1^HNMR. The DCT was released from T*f*-conjugated PLGA NPs in a sustained fashion over a period of 48 h. 

**Figure 6:**
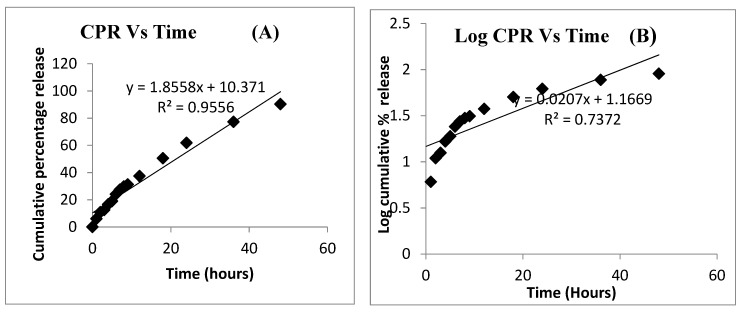

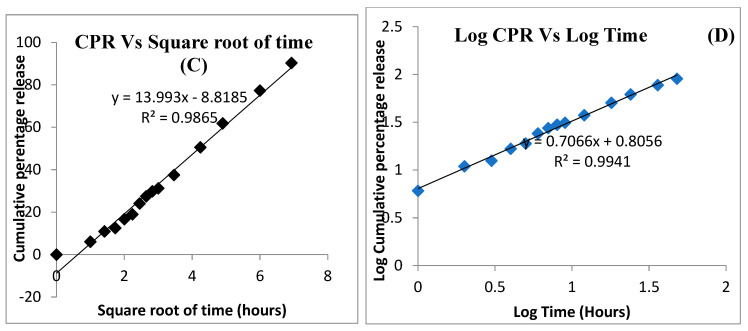
Kinetic assessment of drug release from T*f*-conjugated PLGA NPs using various kinetic models: (**A**) zero-order kinetics, (**B**) first-order kinetics, (**C**) Higuchi’s model, and (**D**) Korsmeyer–Peppa’s model.

Kinetic analysis of the release profile was carried out to determine the exact mechanism of the drug release (Figure 6). The adjustment of the release date for the different mathematical models helped us to describe if the release depended on the drug concentration within the particles (first-order kinetics), whether it was independent on the drug concentration being therefore predictable and at a constant rate (zero-order kinetics) or if it followed a Fickian diffusion. The Higuchi release model describes the release of drugs from an insoluble matrix as a square root of a time-dependent process based on Fickian diffusion and is commonly seen in nanoparticles. In the Korsmeyer–Peppas, for spherical monodispersed particles, a value n − 0.43 is characteristic for Fickian diffusion, but for polydisperse particles to follow Fickian diffusion they should show a lower n value of 0.30, for example [50]. Fickian diffusion stands for the classical diffusion that is controlled by a gradient or differences of concentrations. The zero-order correlation co-efficient value of 0.9556 indicates that the release profile of DCT from T*f*-conjugated PLGA NPs fitted into zero-order kinetics, better than into first-order kinetics. The drug release was diffusion controlled as indicated by the higher *R*^2^ value (0.9865) in the Higuchi model. Since the *n* value (0.7066) obtained from the Korsmeyer–Peppas model or the optimized formulation was between 0.45 and 0.89, the mechanism of drug release from the PLGA NPs was found to follow a non-Fickian diffusion.

The in vitro cytotoxicity of MCF-7 cells incubated with various DCT-loaded T*f*-conjugated PLGA NPs at different drug concentrations was studied by the MTT method. Based on the results shown in Table 6, the various nanoparticle formulations exhibited a dose-dependent cytotoxicity against MCF-7 cells. The cytotoxic potential of DCT-loaded T*f*-conjugated PLGA NPs were much higher than the pure drug, as seen from the IC_50_ values. An IC_50_ value of 7.1 μM/mL was recorded for the pure docetaxel trihydrate. When loaded into nanoparticles, the IC_50_ was 4.392 μM/mL (DCT-loaded T*f*-conjugated PLGA NPs), which was significantly lower than the IC_50_ of unconjugated nanoparticles (6.24 μM/mL, DCT-loaded PLGA NPs). In other words, the conjugation of transferrin improved the efficiency of the chemotherapeutic formulation in comparison to the non-conjugated counterpart. These results also corroborate the conclusion that the drug is indeed entrapped within the PLGA matrix. 

The cell viability of blank nanoparticles was simultaneously examined, and it was found to be between 80% and 90%. This result indicates that blank NPs did not cause evident cytotoxicity. Targeted NPs are taken up by cells via receptor-mediated endocytosis and are not subjected to efflux by P-glycoprotein. The result is a higher cytotoxic effect because the chemotherapeutic compound is efficiently released inside tumor cells. The ANOVA results confirmed that there is a significantly higher cytotoxic effect for transferrin-conjugated nanoparticles than the unconjugated nanoparticles. This result was attributed to the increased targeting potential of the drug-loaded NPs resulting from the ligand binding on the transferrin receptor of the MCF-7 cell line. 

**Figure 7:**
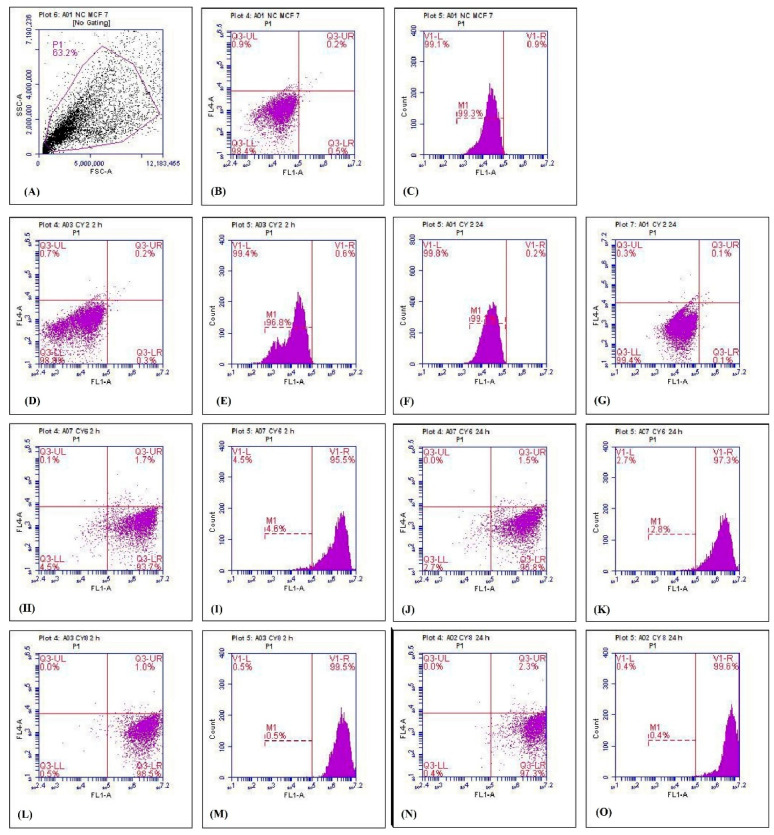
Cellular uptake study using flow cytometry: (**A**) gating, (**B**,**C**) fluorescence of untreated MCF-7 cells, (**D**,**E**) fluorescence of blank nanoparticles at 2 h, (**F**,**G**) fluorescence of blank nanoparticles at 24 h, (**H**,**I**) fluorescence of unconjugated nanoparticles at 2 h, (**J**,**K**) fluorescence of unconjugated nanoparticles at 24 h, (**L**,**M**) fluorescence of DCT-loaded T*f*-conjugated PLGA NPs at 2 h, (**N**,**O**) fluorescence of T*f*-conjugated PLGA NP at 24 h. The *x*- and *y*-axes correspond to forward scatter (FSC) (which measures size) and side scatter (SSC) (which measures internal complexity), respectively. The FL1-area stands for total cell fluorescence.

To evaluate the targeting ability of the nanoparticles, the cellular uptake of coumarin-6-loaded PLGA and PLGA-EDA-Tr nanoparticles by MCF-7 cells was investigated (Figure 7). After an incubation period of 2 h and 24 h, the mean fluorescence was recorded and analyzed. Cells without coumarin were used as a control to show the auto-fluorescence. There was an increase in the fluorescence intensity in a time-dependent fashion with the conjugated nanoparticles compared to the unconjugated nanoparticles. This result was attributed to the presence of transferrin moiety on the outside surface of the nanoparticles enabling their binding with transferrin receptors on the tumor cells and subsequent receptor mediated endocytosis.

The quantitative results for the mean fluorescence of MCF-7 cells 2 h and 24 h after treatment with DCT-loaded T*f*-conjugated PLGA NPs are summarized in Table 7.

The cell cycle analysis of MCF-7 cells was carried out after incubating DCT-loaded T*f*-conjugated PLGA NPs at their respective IC_50_ concentrations. Four distinct phases could be recognized in a proliferating cell population, namely, the G1-, S- (DNA synthesis phase), G2-, and M-phase (mitosis). However, G2- and M-phase, both having an identical DNA content, could not be discriminated based on their differences in DNA content.

**Figure 8:**
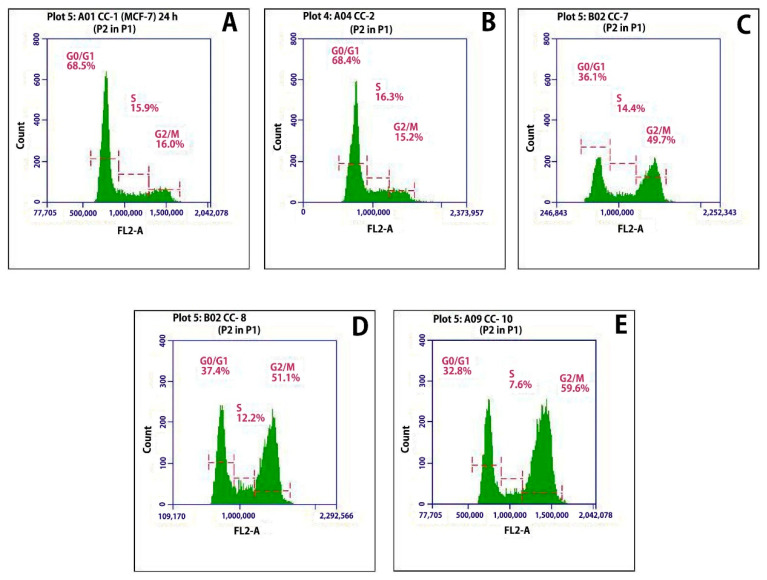
Effect on cell cycle of MCF-7 cells after 24 h of treatment. Control MCF-7 cells (**A**), blank nanoparticles (**B**), docetaxel trihydrate (**C**), non-conjugated nanoparticles (**D**), and DCT-loaded T*f*-conjugated PLGA NPs (**E**). The FL2-area stands for total cell fluorescence.

The control MCF-7 cells showed a cell cycle pattern with 68.5% cells in G0/G1 phase, 15.9% in S phase, and 16.0% cells in G2/M phase (Figure 8A). The blank nanoparticles (Figure 8B) did not show any significant effect on the cell cycle. The pure drug docetaxel trihydrate (Figure 8C) arrested the cell cycle in G2/M phase. The targeted nanoparticles were also able to arrest the cells at G2/M phase with a cell cycle pattern of 32.8% cells in G0/G1 phase, 7.6% in S phase, and 59.6% cells in G2/M phase (Figure 8E).

## 4. Conclusions

Active targeting of the drug molecules with the help of a ligand-conjugated nanoparticles could reduce the undesirable side effects of tumor therapy. In our study, we successfully synthesized PLGA-transferrin conjugate using a novel technique with EDA as a linker. The drug-loaded PLGA nanoparticles were effectively optimized by 3^2^ full factorial design. The FTIR, DSC, PXRD, TEM, particle size, and zeta potential analyses were carried out to characterize the developed nanoparticles. FTIR, DSC, and PXRD analysis confirmed the successful development of ligand-conjugated nanoparticles. The TEM images revealed the particle size and spherical shape of the developed nanoparticles which will limit the systemic distribution of the chemotherapeutic drug in vivo. Particle size distribution by DLS technique showed that the transferrin-conjugated NPs were within an acceptable range. The zeta potentials of the formulations were between −32 and −24 mV, which indicates good colloidal stability due to the repulsive forces. The in vitro release kinetics revealed that the drug release followed a zero-order kinetics by a non-Fickian diffusion mechanism. The effectiveness of developed conjugated NPs was evaluated in vitro against MCF-7 cells. Cytotoxicity studies confirmed that ligand-conjugated NPs are more effective than unconjugated NPs. Cell uptake studies re-confirmed the ligand-mediated active targeting of the formulated NPs. Cell cycle analysis concluded that the anti-cancer activity of all the developed formulation is by arresting the G_2_/M phase in accordance with the literature of docetaxel trihydrate.

## Figures and Tables

**Table 1:** Factors and levels used in the design of experiments.

Independent Variables	Levels		
	−1	0	+1
Phase ratio	1:3	1:4	1:5
Sonication time (min)	6	8	10
Dependent variables	Y_1_—Mean Particle Size		
	Y_2_—Encapsulation Efficiency (EE%)		

**Table 2:** The effect of the chosen independent variables (factors) on the dependent variables (mean particle size and encapsulation efficiency). Loading capacities were also calculated for the 13 formulations.

Formulation Code	Factor 1 Phase Ratio	Factor 2 Sonication Time (min)	Response 1 Mean Particle Size (nm)	Response 2	
				%EE *	%LC
1-Tf-PLGA NPs	1:4	6	283.4	50	25.0
2-Tf-PLGA NPs	1:4	8	280.6	45.6	22.8
3-Tf-PLGA NPs	1:5	10	204.2	32.1	16.1
4-Tf-PLGA NPs	1:4	10	235.5	39.6	19.8
5-Tf-PLGA NPs	1:5	6	230.5	38.4	19.2
6-Tf-PLGA NPs	1:4	8	240.6	44.4	22.2
7-Tf-PLGA NPs	1:4	8	250.2	43.6	21.8
8-Tf-PLGA NPs	1:4	8	247.0	44.8	22.4
9-Tf-PLGA NPs	1:5	8	219.7	36.8	18.4
10-Tf-PLGA NPs	1:4	8	247.8	42.8	21.4
11-Tf-PLGA NPs	1:3	8	370.8	56.8	28.4
12-Tf-PLGA NPs	1:3	6	426.7	59.6	29.8
13-Tf-PLGA NPs	1:3	10	311.0	53.6	26.8

* %EE, percentage of encapsulation efficiency was used as the dependent variable in the factorial design experiment.

**Table 3:** Fit summary for the responses particle size (Y_1_) and entrapment efficiency (Y2) by 3^2^ full factorial design.

Source	Sum of Squares		df		F-Value		*p*-Value Prob > F	
	Y_1_	Y_2_	Y_1_	Y_2_	Y_1_	Y_2_	Y_1_	Y_2_
**Sequential Model Sum of Squares**								
Mean versus Total	9.682 × 10^5^	26,604.74	1	1				
Linear versus Mean	40,378.14	741.1	2	2	24.96	157.99	0.0001	0.0001
2FI * versus Linear	1998.09	0.022	1	1	2.95	8.642 × 10^−3^	0.1198	0.9280
Quadratic versus 2FI	4877.38	10.71	2	2	14.08	2.95	0.0035	0.1179
Cubic versus Quadratic	177.92	6.63	2	2	0.43	2.72	0.6725	0.1587
Residual	1034.31	6.09	5	5				
**Lack of Fit Tests**								
Linear	7101.59	18.78	6	6	4.80	2.68	0.0754	0.1796
2FI *	5103.50	18.76	5	5	4.14	3.21	0.0967	0.1406
Quadratic	226.12	8.05	3	3	0.31	2.30	0.8211	0.2194
Cubic	48.20	1.42	1	1	0.20	1.22	0.6812	0.3320
Pure Error	986.11	4.67	4	4				
**Model Summary Statistics**								
**Source**	***R*-Squared**		**Adjusted *R*-Squared**		**Predicted *R*-Squared**		**PRESS**	
	**Y1**	**Y2**	**Y1**	**Y2**	**Y1**	**Y2**	**Y1**	**Y2**
Linear	28.44	1.53	0.7998	0.9632	0.6572	0.9476	16,612.71	40.09
2FI *	26.01	1.61	0.8325	0.9591	0.5742	0.9166	20,636.95	63.73
Quadratic	13.16	1.35	0.9571	0.9715	0.9261	0.8901	3579.85	83.99
Cubic	14.38	1.10	0.9488	0.9809	0.8547	0.7752	7040.67	171.85

2FI *, sequential sum of squares for the two-factor interaction terms.

**Table 4:** Analysis of variance of responses of particle size (Y1) and encapsulation efficiency (Y2).

Source	Sum of Squares	df	Mean Square	F-Value	*p*-Value Prob > F	-
**Response Y1: Particle Size**						
Model	47,253.61	5	9450.72	54.57	<0.0001	significant
A-phase Ratio	34,367.80	1	34,367.80	198.46	<0.0001	
B-sonication Time	6010.33	1	6010.33	34.71	0.0006	
AB	1988	1	1998.09	11.54	0.0115	
A^2^	3982.76	1	3982.76	23.0	0.0020	
B^2^	13.06	1	13.06	0.075	0.7916	
Residual	1212.24	7	173.18			
Lack of Fit	226.12	3	75.37	0.31	0.8211	not significant
Pure Error	986.11	4	246.53			
Cor Total	48,465.84	12				
**Response Y2: Encapsulation Efficiency**						
Model	741.1	2	370.55	157.99	<0.0001	significant
A-phase Ratio	655.22	1	655.22	279.36	<0.0001	
B-sonication Time	85.88	1	85.88	36.62	0.0001	
Residual	23.45	10	2.35			
Lack of Fit	18.78	6	3.13	2.68	0.1796	not significant
Pure Error	4.67	4	1.17			
Cor Total	764.55	12				

**Table 5:** Composition of the optimized formulation of DCT-loaded T*f*-conjugated PLGA NPs and predicted and actual responses.

Factors	Level	Average Particle Size (nm)		Average Encapsulation Efficiency (%)	
Phase ratio	1:4.7	Predicted	Real	Predicted	Real
Sonication time (min)	10	206.2	210.6 ± 2.7	34.1	36.1 ± 2.3

**Table 6:** In vitro cytotoxicity assay in MCF-7 cell lines.

Compound Name	Concentration (μM/mL)	Mean Cell Death	SEM	IC_50_ (μM/mL)
Docetaxel	0.25	16.5	2.9	7.097
	0.5	23.0	1.1	
	1	41.6	1.0	
	2	51.0	0.7	
DCT-loaded PLGA NPs	0.25	25.5	0.9	6.24
	0.5	36.4	3.6	
	1	43.8	0.6	
	2	53.0	1.0	
DCT-loaded T*f*-conjugated PLGA NPs	0.25	23.0	1.1	4.392
	0.5	41.6	1.0	
	1	51.0	0.7	
	2	56.6	0.8	
Blank NPs	100	18.2	7.2	800 μg/mL
	200	16.0	4.0	
	400	19.3	3.9	
	800	15.8	10.4	

**Table 7:** Mean fluorescence analysis of MCF-7 cells 2 h and 24 h after treatment with blank NPs, DCT-loaded PLGA NPs, and DCT-loaded T*f*-conjugated PLGA NPs.

Samples	Mean Fluorescence	
	2 h	24 h
Blank NPs	27,294.1	32,914.2
DCT-loaded PLGA NPs	2,930,523.01	3,085,163.04
DCT-loaded T*f*-conjugated PLGA NPs	4,153,708.4	7,550,576.4

## Supplementary Materials

The following are available online at https://www.mdpi.com/2073-4360/11/11/1905/s1, Figure S1. FTIR spectrum of docetaxel trihydrate (DCT) (upper), of physical mixture of docetaxel trihydrate, transferrin (T*f*) and PLGA (middle), and of DCT-loaded T*f*-conjugated PLGA NPs (lower); Figure S2. DSC thermogram of PLGA (A), transferrin (B), docetaxel trihydrate (C), their physical mixture (D) and of DCT-loaded T*f*-conjugated PLGA NPs (E); Figure S3. (A) X-ray diffraction pattern of docetaxel trihydrate, (B) physical mixture of docetaxel trihydrate, transferrin and PLGA, and of (C) DCT-loaded T*f*-conjugated PLGA NPs. Figure S4. (A) Particle size distribution and average particle size and (B) zeta potential of DCT-loaded Tf-conjugated PLGA NPs.
