# Correction: Jose et al. Transferrin-Conjugated Docetaxel–PLGA Nanoparticles for Tumor Targeting: Influence on MCF-7 Cell Cycle. *Polymers* 2019, *11*, 1905

**DOI:** 10.3390/polym15010189

**Published:** 2022-12-30

**Authors:** Sajan Jose, Thomas A. Cinu, Rosmy Sebastian, M. H. Shoja, N. A. Aleykutty, Alessandra Durazzo, Massimo Lucarini, Antonello Santini, Eliana B. Souto

**Affiliations:** 1 Department of Pharmaceutical Sciences, Mahatma Gandhi University, Cheruvandoor Campus, Ettumanoor 686631, India; 2 CEB-Centre of Biological Engineering, University of Minho, Campus de Gualtar, 4710-057 Braga, Portugal; 3 College of Pharmaceutical Sciences, Manipal University, Manipal 576104, India; 4 Caritas College of Pharmacy, Kottayam 686630, India; 5 CREA—Research Centre for Food and Nutrition, Via Ardeatina 546, 00178 Rome, Italy; 6 Department of Pharmacy, University of Napoli Federico II, Via D. Montesano 49, 80131 Napoli, Italy; 7 Department of Pharmaceutical Technology, Faculty of Pharmacy, University of Coimbra (FFUC), Pólo das Ciências da Saúde, 3000-548 Coimbra, Portugal

In the original publication [1], there was a mistake in Figure 7. Figure 7B,C (Untreated MCF7 Cells) and Figure 7D,E (Blank nano particle @2h) have, in fact, very low uptake and have almost the same data. However, when changing to drug-loaded nanoparticles and transferrin-conjugated drug-loaded nanoparticles, the uptake increases. When building the combined image, the panels D and E were misplaced. The correct Figure 7 appears below. The authors state that the scientific conclusions are unaffected. This correction was approved by the Academic Editor. The original publication has also been updated.

## Figures and Tables

**Figure 7:**
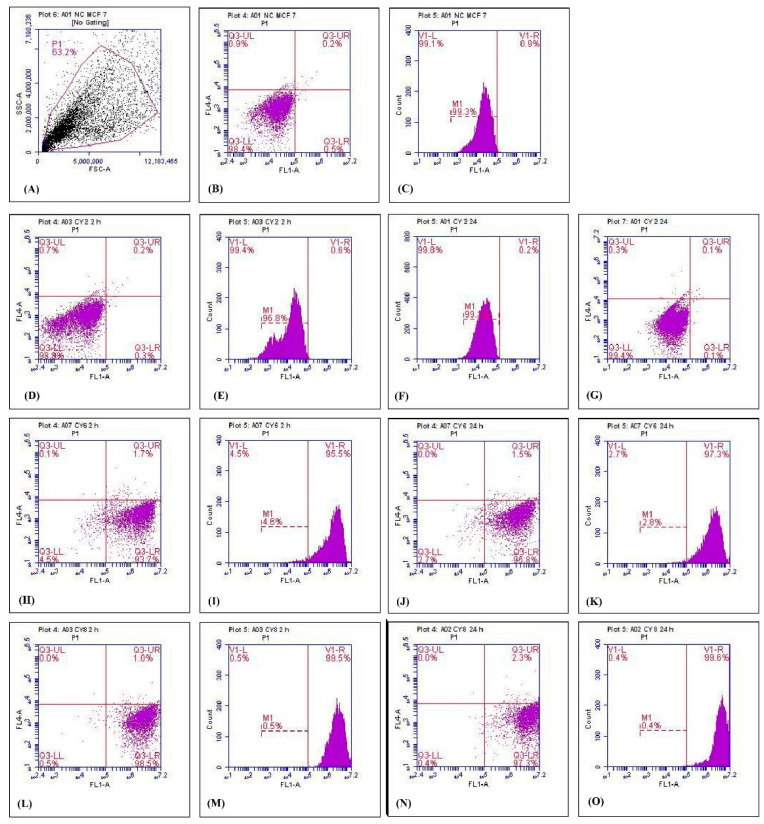
Cellular uptake study using flow cytometry: (**A**) gating, (**B**,**C**) fluorescence of untreated MCF-7 cells, (**D**,**E**) fluorescence of blank nanoparticles at 2 h, (**F**,**G**) fluorescence of blank nanoparticles at 24 h, (**H**,**I**) fluorescence of unconjugated nanoparticles at 2 h, (**J**,**K**) fluorescence of unconjugated nanoparticles at 24 h, (**L**,**M**) fluorescence of DCT-loaded T*f*-conjugated PLGA NPs at 2 h, (**N**,**O**) fluorescence of T*f*-conjugated PLGA NP at 24 h. The *x*- and *y*-axes correspond to forward scatter (FSC) (which measures size) and side scatter (SSC) (which measures internal complexity), respectively. The FL1-area stands for total cell fluorescence.
